# Value of Cardiac Troponin, Myoglobin Combined with Heart-type Fatty Acid-binding Protein Detection in Diagnosis of Early Acute Myocardial Infarction

**DOI:** 10.12669/pjms.39.6.7101

**Published:** 2023

**Authors:** Jian hua Sun, Xiao kun Liu, Xiao wei Xing, Yang Yang, Hui hong Xuan, Bin bin Fu

**Affiliations:** 1 Jian hua Sun, Department of Cardiology, Tangshan Workers’ Hospital, Tangshan, 063000, Hebei, P.R. China; 2 Xiao kun Liu, Department of Cardiology, Tangshan Workers’ Hospital, Tangshan, 063000, Hebei, P.R. China; 3 Xiao wei Xing, Department of Cardiology, Tangshan Workers’ Hospital, Tangshan, 063000, Hebei, P.R. China; 4 Yang Yang, Department of Cardiology, Tangshan Workers’ Hospital, Tangshan, 063000, Hebei, P.R. China; 5 Hui hong Xuan, Department of Cardiology, Tangshan Workers’ Hospital, Tangshan, 063000, Hebei, P.R. China; 6 Bin bin Fu, Department of Cardiology, Tangshan Qianxi people’s Hospital, Tangshan, 064303, Hebei, P.R. China

**Keywords:** Ctn, Myo, H-FABP, Combined detection, Acute myocardial infarction, Diagnosis

## Abstract

**Objective:**

To evaluate the value of cardiac troponin(cTn), myoglobin(Myo) combined with heart-type fatty acid-binding protein(H-FABP) detection in the diagnosis of early acute myocardial infarction(AMI).

**Methods:**

This study was a clinical comparative study. Eighty patients with AMI hospitalized in Tangshan Workers’ Hospital were selected as study group, and another 80 individuals receiving normal physical examination were selected as control group from September 20, 2021 to September 20, 2022. The concentrations of cTn, Myo and H-FABPP, diagnostic indicators, the sensitivity and specificity of combined diagnosis, as well as the diagnostic efficacy for AMI were compared between the two groups.

**Results:**

The levels of cTn, Myo and H-FABPP in the study group were significantly higher than those in the control group(*P*= 0.00). Multivariate logistic regression analysis showed that cTn, Myo and H-FABP were all relevant indicators for AMI. H-FABP alone has better diagnostic efficacy for AMI. The area under the curve of their combined detection, the specificity, and the sensitivity were higher than those of cTn, Myo and H-FABP alone, indicating that their combined application has the best diagnostic efficiency. cTn, Myo and H-FABP levels were positively correlated with Glu, TC, LDL-C and hs-CRP levels(*P*< 0.01), while negatively correlated with HDL level(*P*< 0.01).

**Conclusions:**

The combined detection of cardiac markers such as cTn, Myo and H-FABP presents higher sensitivity and specificity in the diagnosis of AMI compared with any single detection, and can provide better data support for the definite diagnosis of AMI, with high clinical application value.

## INTRODUCTION

Acute myocardial infarction(AMI) is defined as severe insufficient blood supply to the coronary artery caused by coronary atherosclerosis or other reasons, leading to myocardial ischemia and hypoxia, and then ischemic necrosis, and it is a common cause of death in the elderly.^1^ Its main clinical symptom is sudden and persistent precordial crushing pain, accompanied by radiating pain in the left shoulder, left forearm or left fingers, which cannot be alleviated by rest or oral nitrates, and has dynamic changes in electrocardiography(ECG) and laboratory biochemical examination.^2^ Patients with mild or atypical symptoms only feel chest tightness or even have no symptoms, while severe ones can present sudden left heart failure(HF), arrhythmia, shock and even sudden death in a short time.

The condition of patients with AMI is dangerous and changeable, and they are prone to severe complications. Although percutaneous coronary intervention (PCI) has made progress and been widely used, the mortality of patients with AMI is still high, and the treatment options are limited.^3^ Therefore, early correct diagnosis and effective treatment are extremely important in reducing the mortality and improving the prognosis of patients with AMI.^4^ For the diagnosis of MI, dynamic changes of ECG and myocardial enzymology are commonly used. However, for AMI patients with atypical clinical symptoms and nonspecific changes in ECG, the detection of myocardial markers has become an important basis for diagnosis.^5^

Rapid detection of cardiac markers is one of the most important examinations for the early diagnosis of AMI.^6^ At present, great progress has been made in the research on MI markers in AMI. Myoglobin (Myo) and cardiac troponin (cTn) are widely used in clinical practice. Among them, Myo appears early, and its concentration increases about 2-3 hours after chest pain, but its specificity is poor. Although cTn has high specificity, its concentration increases 6-8 hours after chest pain.^7^ Heart-type fatty acid-binding protein (H-FABP) is one of the highly sensitive biochemical indicators for early diagnosis of AMI, which has been widely studied at home and abroad.^8^ In this study, the combined detection of cardiac markers such as cTn, Myo and H-FABP was carried out, and the advantages of single detection and different combinations of cardiac markers were compared, achieving certain results.

## METHODS

This was a clinical comparative study. A total of 80 patients with early AMI hospitalized in Tangshan Workers’ Hospital were selected as study group, including 53 males and 27 females, aging 63-78 years, with an average age of 70.267± 7.54 years. Additionally, 80 healthy individuals receiving physical examination in the same period were selected as control group, including 58 males and 22 females, with an age of 68-80 years (average, 72.31±7.39 years) from September 20, 2021 to September 20, 2022. The general data showed no statistical differences between the two groups, suggesting comparability (Table-I).

**Table-I:** Comparison and analysis of general data between the two groups (*χ̅*±*S*) n = 80.

Indicator	Study group	Control group	t/χ^2^	P
Male(n %)	53(66.3%)	58(72.5%)	0.74	0.39
Age(year)	70.26±7.54	72.31±7.39	1.74	0.08
Hypertension(n %)	23(28.8%)	25(31.2%)	0.12	0.73
Diabetes(n %)	15(18.8%)	19(23.8%)	0.60	0.44
Smoking history(n %)	17(21.2%)	13(16.3%)	0.66	0.42
Drinking history(n %)	25(31.2%)	27(33.7%)	0.11	0.74
BMI(kg/m^2^)	24.63±2.52	24.21±2.47	1.06	0.29

P > 0.05.

### Ethical Approval

The study was approved by the Institutional Ethics Committee of Tangshan Workers’ Hospital (No.: GRYY-LL-KJ2022-047; June 07,2022), and written informed consent was obtained from all participants.

### Inclusion criteria:

- Meeting two of the diagnostic criteria of AMI.^9^
- The medical history of chest pain, which could not be alleviated by rest or oral nitroglycerin tablets.
- Dynamic changes in ECG after onset: (1) increased levels of cardiac markers such as myocardial zymogram in laboratory examination, (2)onset time < 24 hours, (3)age < 80 years.
- No mental illness or cognitive impairment, and able to cooperate with the research work.
- Complete clinical data.
- The patients and their families signed the consent form and were able to cooperate the research work.

### Exclusion criteria:

- Complicated with other cardiovascular diseases.
- Complicated with autoimmune diseases.
- Complicated with malignant tumors.
- Complicated with severe dysfunction of the liver, kidney, brain and other important organs.
- Complicated with myocarditis or hyperthyroidism.
- Mental illness or other reasons leading to inability to cooperate the study.

After admission, 10 ml of fasting venous blood was collected from all patients in the study group. After standing for 10 minutes, the fasting venous blood was centrifuged at 2000 r/minutes for five minutes, and the supernatant was collected for subsequent detection. (1)Serum cTn and Myo levels were measured in the two groups using enzyme-linked immunosorbent assay (ELISA). (2)The level of serum H-FABPP was detected by Beckmann full-automatic biochemical analyzer. (3)The levels of blood glucose, blood lipid and high-sensitivity C-reactive protein (Hs-CRP) were determined using Hitachi 7600 full-automatic biochemical analyzer. All detection were carried out in strict accordance with the instructions of the kits.

### Observation indicators

(1)The levels of cTn, Myo and H-FABPP in the two groups were compared and analyzed. (2)The specificity, accuracy and sensitivity of blood cTn, Myo and H-FABPP combined diagnosis and single diagnosis were compared and analyzed. (3)The correlations of cTn, Myo and H-FABPP with blood glucose, blood lipid and Hs-CRP were analyzed.

### Statistical Analysis

All data were statistically analyzed using SPSS 20.0. The measurement data were expressed as (*χ̅*±*S*), and the enumeration data were expressed as absolute or constituent ratio. Two-group independent sample t-test was used for inter-group analysis, and χ^2^ test for rate comparison. The influencing factors were analyzed by logistic regression. The area under the curve (AUC) and the optimal diagnostic threshold of cTn, Myo, H-FABP and their combined detection were analyzed, and the sensitivity, specificity and misdiagnosis rate were calculated. The correlations were analyzed using Pearson correlation coefficient. *P*< 0.05 was considered as statistically significant.

## RESULTS

The levels of cTn, Myo and H-FABPP showed differences between the study group and the control group, and were significantly higher in the study group than those in the control group(*P*= 0.00)(Table-II). With cTn, Myo and H-FABP as independent variables, multivariable logistic regression analysis was carried out after assignment, revealing α_input_ = 0.05 and α_output_ = 0.10, which suggested that cTn (*P*= 0.002), Myo (*P*= 0.004) and H-FABP (*P*= 0.001) were all relevant indicators for AMI (Table-III).

**Table-II:** Differences in cTn, Myo and H-FABP between study group and control group(*χ̅*±*S*) n = 80.

Indicator	Study group	Control group	t	p
cTn(ng/L)	4.79±1.37	1.53±0.67	19.12	0.00
Myo(ug/L)	87.65±14.74	58.46±12.43	36.73	0.00
H-FABP(ng/ml)	42.57±10.32	3.47±0.26	33.87	0.00

P < 0.05.

**Table-III:** Multivariable logistic regression analysis of cTn, Myo and H-FABP in patients with AMI n = 80.

Relevant variables	β	SE	Waldχ^2^	P	OR	95.0% CI
cTn(ng/L)	2.783	0.962	8.470	0.002	14.583	12.531~17.804
Myo(ug/L)	2.057	0.524	7.362	0.004	3.981	1.433~5.827
H-FABP(ng/ml)	3.581	0.672	8.924	0.001	2.327	1.763~4.639

*P < 0.05.

The diagnostic value of cTn, Myo, H-FABP and their combination in AMI (Table-IV) demonstrated that the AUC of H-FABP was 89.6%, the sensitivity was 87.538%, and the specificity was 93.276%, which were higher than those of cTn and Myo, suggesting that H-FABP alone has better diagnostic efficacy for AMI. The AUC of their combined detection was 93.7%, the specificity was 95.843%, and the sensitivity was 93.468%, which were higher than those of cTn, Myo and H-FABP alone, indicating that their combined application has the best diagnostic efficiency.

**Table-IV:** Diagnostic significance of cTn, Myo, H-FABP and their combination in AMI n = 80.

Indicator	Critical value	Sensitivity %	Specificity %	Missed diagnosis rate %	Misdiagnosis rate %	AUC	95.0% CI
cTn(ng/L)	13.472	85.473	89.047	14.572	10.953	0.874	0.815~0.890
Myo(ug/L)	17.560	80.216	81.018	19.784	18.982	0.840	0.772~0.869
H-FABP(ng/ml)	20.835	87.538	93.276	12.620	6.724	0.896	0.842~0.953
Combined detection		93.468	95.843	6.532	4.157	0.937	0.804~0.972

The correlation analysis of cTn, Myo and H-FABP with biochemical indicators in patients with AMI revealed that cTn, Myo and H-FABP levels were positively correlated with Glu, TC, LDL-C and hs-CRP levels (*P*< 0.01), while negatively correlated with HDL level (*P*< 0.01). However, no significant correlations were found with UA, Cr or TG level (*P*> 0.05) (Table-V).

**Table-V:** Correlation analysis of cTn, Myo and H-FABP with biochemical indicators in patients with AMI (*χ̅*±*S*) n = 80.

Biochemical indicator	H-FABP		cTn		Myo	

	r	p	r	p	r	p
Glu*	0.782	0.00	0.643	0.00	0.607	0.00
UA	0.036	0.37	0.055	0.26	0.028	0.43
Cr	0.105	0.75	0.089	0.88	0.106	0.29
TC*	0.576	0.01	0.611	0.00	0.582	0.00
TG	0.045	0.62	0.038	0.39	0.107	0.53
HDL*	-0.372	0.00	-0.426	0.00	-0.370	0.00
LDL*	0.658	0.00	0.673	0.00	0.439	0.00
Hs-CRP*	0.317	0.02	0.364	0.01	0.329	0.01

* P < 0.05.

## DISCUSSION

We used cTn, Myo and H-FABP to evaluate early AMI. The results confirmed that cTn (*P*= 0.002), Myo (*P*= 0.004) and H-FABP (*P*= 0.001) were all relevant indicators for AMI. The sensitivity of H-FABP was 87.538%, and the specificity was 93.276%, which were higher than those of cTn and Myo, suggesting that H-FABP alone has better diagnostic efficacy for AMI. The AUC of their combined detection was 93.7%, the specificity was 95.843%, and the sensitivity was 93.468%, which were higher than those of cTn, Myo and H-FABP alone, indicating that their combined application has the best diagnostic efficiency. The correlation analysis of cTn, Myo and H-FABP with biochemical indicators in patients with AMI revealed that cTn, Myo and H-FABP levels were positively correlated with Glu, TC, LDL-C and hs-CRP levels (*P*< 0.01), while negatively correlated with HDL level (*P*< 0.01).

The pathophysiological mechanism of AMI lies in coronary atherosclerosis which leads to coronary stenosis, thrombosis which causes occlusion, or myocardial necrosis caused by ischemia and hypoxia of some myocardial cells. Common causes include fatigue, emotional irritability, overeating, hypertension, hyperlipidemia and other basic medical histories.^10^ With the aging of the population and the changes in living and eating habits, the incidence of AMI has increased year by year all over the world in recent years.^11^ AMI is complex and dangerous, with high mortality, which seriously affects people’s life and health.^12^

With the increasing maturity of new treatment methods such as thrombolysis, reperfusion and intervention, the therapeutic effect of AMI has been significantly improved. However, about 25% patients still lack typical clinical symptoms and ECG changes in early onset, resulting in delayed diagnosis and treatment, and missing the best treatment time.^13^ Therefore, the detection of cardiac markers, especially selecting the best detection indicator or combined detection in the early stage, is of great significance to provide support for clinical diagnosis and treatment.^14^ The ideal cardiac markers should have the advantages of rapidity, low cost and accuracy. In addition, they are only expressed in myocardial tissue and can be detected after myocardial injury, with high sensitivity and specificity.^15^

The content of Myo in normal human serum is very low. After the onset of AMI, Myo is rapidly released into the blood, its serum concentration begins to rise 1~2 hours after the onset of symptoms, reaches the peak after 5~10 hours, and begins to return to the baseline level one d later.16 After myocardial injury, Myo can be quickly detected in the blood, so it is often used for early diagnosis of patients with AMI and detection of the efficacy of thrombolytic and reperfusion therapy, as well as considered as one of the most sensitive indicators for early diagnosis of AMI.^17^ Asl et al.^18^ believe that Myo has a short window period and can be quickly cleared by the kidney. Therefore, Myo can also be used to judge whether thrombolytic therapy is successful in patients with AMI. Sharma et al.^19^ believe that the detection sensitivity of Myo in patients with AMI is higher than CK-MB and troponin, but Myo still exists in the skeletal muscle, so its specificity is not high.

Early detection of cTn is very important for the diagnosis of AMI(AMI) and HF.^20^ cTn has been widely used as a cardiac marker. The coding of cTn gene is different from that of troponin gene in the skeletal muscle. Troponin only exists in myocardial cells, so its specificity is higher than Myo, and it has become an important indicator for the early diagnosis of cardiovascular diseases in recent years.^21^ Troponin is unique to myocardial cells. When myocardial injury and cell necrosis occur, it is quickly released into the blood through the intact cell membrane. Troponin lasts for a long time in the blood after AMI.

The specificity of troponin is not affected by skeletal muscle injury, strenuous exercise and renal diseases, and its specificity and sensitivity are both significantly higher than those of creatine kinase isoenzyme.^22^ In addition, serum troponin level does not change with age, gender, location of myocardial injury, and type of thrombolytic drugs.^23^ However, its rise time in the peripheral blood is late, so the clinical diagnosis of AMI needs the combined detection of cTn and Myo, but the diagnostic ability is relatively limited.^24^

H-FABP is a small-molecule protein (14~15 kD), which has strict organ specificity and massively exists in the cytoplasm of myocardial cells.^25^ It has been found that the content of H-FABP in normal human plasma is very low, but when the body has myocardial cell damage, it can be quickly released into the blood and its level presents a sharp upward trend, as well as its release is positively correlated with the range of myocardial injury.^26^ Goel et al.^27^ have confirmed that for AMI within three hours of onset, its sensitivity and specificity are both higher than traditional myocardial enzymes, and it can predict the area of MI earlier than traditional myocardial enzymes. Therefore, it is believed that H-FABP can replace traditional myocardial enzymes to predict and evaluate myocardial injury.

For the early diagnosis of MI, ideal cardiac markers should have the characteristics of high sensitivity, accuracy and timeliness in addition to tissue specificity. However, there is no marker for myocardial injury meeting the above requirements. Therefore, the accuracy of single cardiac marker detection in the diagnosis of AMI is low, and the combined diagnosis of multiple indicators can improve its diagnostic rate.^28^

### Limitations

It includes sample size is small, the follow-up time is short, and there is still a lack of some research contents, such as the predictive value of indicator levels on prognosis. Therefore, whether there are more sensitive combined indicator applications needs more in-depth research with larger sample size and longer observation time in the future.

## CONCLUSION

In conclusion, the combined detection of cardiac markers such as cTn, Myo and H-FABP presents higher sensitivity and specificity in the diagnosis of AMI compared with any single detection, and can provide better data support for the definite diagnosis of AMI, with high clinical application value.

### Authors’ Contributions:

**JS** and **XL:** designed this study and prepared this manuscript, and are responsible and accountable for the accuracy and integrity of the work. **XX** and **YY:** collected and analyzed clinical data. **HX** and **BF:** Reviewed and significantly revised this manuscript.
